# Primary Nephrotic Syndrome in Adults as a Risk Factor for Pulmonary Embolism: An Up-to-Date Review of the Literature

**DOI:** 10.1155/2014/916760

**Published:** 2014-04-16

**Authors:** Aibek E. Mirrakhimov, Alaa M. Ali, Aram Barbaryan, Suartcha Prueksaritanond, Nasir Hussain

**Affiliations:** ^1^Department of Internal Medicine, Saint Joseph Hospital, 2900 N. Lake Shore, Chicago, IL 60657, USA; ^2^College of Physicians and Surgeons, Columbia University, New York-Presbyterian Hospital, New York, NY 10034, USA

## Abstract

Patients with nephrotic syndrome are at an increased risk for thrombotic events; deep venous thrombosis, renal vein thrombosis, and pulmonary embolism are quite common in patients with nephrotic syndrome. It is important to note that nephrotic syndrome secondary to membranous nephropathy may impose a greater thrombotic risk for unclear reasons. Increased platelet activation, enhanced red blood cell aggregation, and an imbalance between procoagulant and anticoagulant factors are thought to underlie the excessive thrombotic risk in patients with nephrotic syndrome. The current scientific literature suggests that patients with low serum albumin levels and membranous nephropathy may benefit from primary prophylactic anticoagulation. A thorough approach which includes accounting for all additional thrombotic risk factors is, therefore, essential. Patient counseling regarding the pros and cons of anticoagulation is of paramount importance. Future prospective randomized studies should address the question regarding the utility of primary thromboprophylaxis in patients with nephrotic syndrome.

## 1. Introduction

Nephrotic syndrome (NS) is characterized by proteinuria of ≥3.5 g/24 hours, albuminemia <3.0 g, peripheral edema, hyperlipidemia, lipiduria, and increased thrombotic risk [1, 2]. The etiology of NS is divided into primary NS and secondary NS. Furthermore, secondary causes of NS can be subdivided into NS-related systemic diseases and NS related to medication use. Common primary causes of NS are focal segmental glomerulosclerosis (FSGS), membranous nephropathy (MN), and minimal change disease (MCD) (after excluding identifiable causes such as cancer, systemic diseases, and medications) [2]. Common causes of NS secondary to systemic diseases are diabetes mellitus, systemic lupus erythematosus, multiple myeloma, amyloidosis, malignancy, and infections [2]. Pamidronate, nonsteroidal anti-inflammatory drugs, penicillamine, and gold compounds are among the most commonly implicated medications in the development of NS [2].

Primary NS is a relatively rare entity compared to NS secondary to systemic diseases, particularly diabetes mellitus [2]. FSGS and MN are the more common pathological forms of NS in adults, whereas MCD is more common in the pediatric population. Some cases of immunoglobulin A nephropathy (IgAN) and membranoproliferative glomerulonephritis may clinically present with NS [2–4]. It is important to note that renal biopsy is essential in making a diagnosis and in certain diseases (such as systemic lupus erythematosus) it guides the therapy [5].

Pulmonary embolism (PE) is a serious complication of deep venous thrombosis (DVT), with a significant morbidity and mortality [6, 7]. Common risk factors for the development of DVT and PE are prolonged immobilization, recent surgery, cancer, cardiac disease, autoimmune disease (such as inflammatory bowel disease), and prior history of DVT/PE and hypercoagulable conditions (such as NS and antiphospholipid antibody syndrome) [8, 9].

The clinical presentation of PE can range from asymptomatic to tachypnea and tachycardia to cardiovascular collapse and death [6]. Moreover, episodes of PE can have long-term consequences such as chronic thromboembolic pulmonary hypertension (CTEPH) [10]. High clinical suspicion is, therefore, paramount in the diagnosis of PE.

The goal of this paper is to summarize the scientific data regarding the impact of primary NS on the risk of PE. We will review the current understanding of the pathophysiology of increased thrombotic risk among patients with NS. Second, clinical data and thrombosis predictors will be reviewed. Third, we will review the current data on the utility of prophylactic anticoagulation. However, we will not provide a detailed review on the clinical presentation, imaging, and treatment of PE, since these topics are out of the paper's scope and can be easily found elsewhere [6].

## 2. Why Do Patients with Nephrotic Syndrome Are at Increased Risk for Thrombotic Events?

NS on a tissue level represents damage to the glomerulus, with resultant dysfunction and permeability to various endogenous substances which are not filtered through the glomerular membrane under normal physiologic conditions [2]. Most features of NS are directly related to the increased glomerular permeability, for example, proteinuria, hypoalbuminemia, and lipiduria. The hypercoagulable state seen in patients with primary NS is believed to be secondary to glomerular pathology. However, patients with malignancy-related NS are likely to represent a group who is at even greater risk for thrombotic events due to underlying cancer [11]. Below, we will review the current concepts of increased thrombotic risk among patients with primary NS.

Patients with NS have increased platelet reactivity and often thrombocytosis [12–15]. The pathophysiology of platelet hyperactivity is not entirely understood, but several key factors are believed to contribute. First, it is well known that thromboxane A2 (TxA2) is a major promoter of platelet activation and clot formation [16]. Arachidonic acid (AA), which is a precursor for TxA2 synthesis, is released from cells in a constant fashion [17]. Albumin binds AA, thus, making it unavailable for platelet metabolism and conversion into TxA2; therefore, TxA2 levels are increased in patients with NS because of hypoalbuminemia, thereby favoring clot formation and platelet hyperactivity [18–21]. Second, elevated fibrinogen levels seen in patients with NS can promote platelet aggregation [22]. Third, elevated cholesterol (commonly seen in patients with NS) can promote platelet aggregation [23, 24]. Finally, patients with NS have increased levels of Von Willebrand factor (vWf) and decreased red blood cell membrane flexibility, which promote platelet adhesion [22].

It is interesting to note that the aggregation of red blood cells may be increased in patients with NS and, thus, may contribute to thrombogenesis [25, 26]. This phenomenon is believed to be secondary to hypoalbuminemia, intravascular volume depletion, red blood cell dehydration (secondary to hypernatremia), and increased fibrinogen levels.

At the level of the coagulation system, several events lead to the thrombotic risk seen in patients with NS. First, antithrombin III (ATIII) levels are decreased in patients with NS. ATIII is a potent endogenous antithrombotic substance and a major factor responsible for the clinical activity of heparin, which targets several coagulation factors such as factor II, factor VII, factor IX, factor X, and factor XII [27]. Urinary loss of ATIII secondary to glomerular membrane permeability is believed to be a principal source of decreased ATIII levels in patients with NS [28–30]. Another mechanism for decreased levels of ATIII observed in patients with NS can be constantly ongoing subclinical thrombosis with the consumption of ATIII [31]. Second, protein S activity may be impaired in patients with NS. Protein S is an essential vitamin K dependent cofactor of protein C that is involved in the inactivation of coagulation factor V and factor VII [32]. It is interesting to note that protein S levels in patients with NS may be increased [33]. However, protein S is present in two forms, free (active) and protein-bound (inactive), with the active form being lost in the urine of patients with NS compared to the inactive form [34, 35]. Therefore, most of the measured protein S in patients with NS is the protein-bound form, which is not physiologically active. However, certain anticoagulation factors such as protein C and tissue factor pathway inhibitor are preserved in patients with NS, which is believed to compensate for the procoagulant state seen in NS  [36, 37]. However, these results were observed in pediatric patients and it is not clear whether they can be extrapolated to adult patients with NS. Nevertheless, the above-mentioned compensatory increase in protein C and tissue factor pathway inhibitor is inadequate to counteract the increased risk of thrombosis in patients with NS.

On the other hand, fibrinogen levels are increased in patients with NS [38]. This increase is believed to be mediated by hypoalbuminemia which in turn increases hepatic fibrinogen synthesis. As mentioned above, fibrinogen enhances platelet reactivity and red blood cell aggregation [22, 39]. Furthermore, patients with NS have greater levels of coagulation factor V, factor VII, and alpha-2 macroglobulin, which is believed to be secondary to upregulated production [39]. However, levels of coagulation factor XI were reported to be decreased in children with NS [40]. Decreased levels of factor XI may be protective against thrombotic events since elevated level of factor XI portends a greater risk of thrombosis in patients without NS [41].

Patients with NS have reduced fibrinolytic activity [42] and increased urinary losses of plasmin, which is a key fibrinolytic protein [43]. In addition, increased levels of lipoprotein a (Lpa) in patients with NS may further counteract fibrinolytic activity [44]. Moreover, patients with NS have increased levels of plasminogen activator inhibitor-1, which is a natural inhibitor of the conversion of plasminogen to plasmin [45]. It is essential to note that fibrin clots in patients with NS may be more resistant to fibrinolysis because of lower thrombus porosity [46]. Furthermore, from a theoretical point of view, it is possible that diuretic use (used to mitigate NS associated body edema) in patients with NS may potentiate hemoconcentration, which, in turn, will promote clot formation.

**Figure 1:**
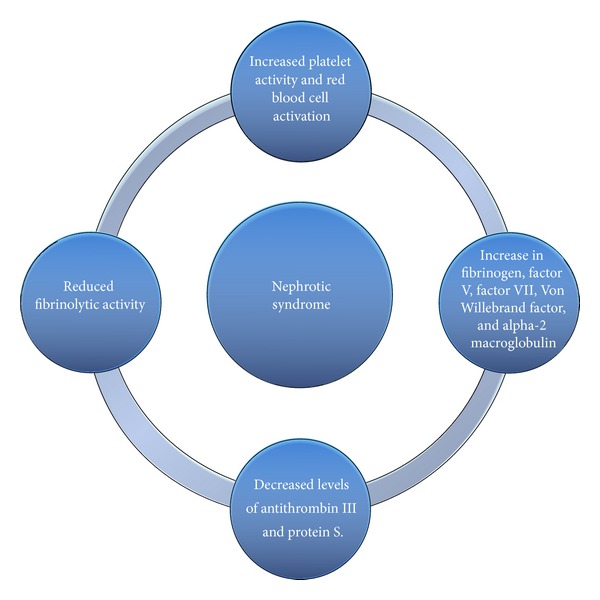
A simplified sketch on the pathogenesis of NS-related thrombotic risk.

Future studies should explore the mechanisms responsible for the increased thrombotic risk seen in NS secondary to MN. A simplified overview of the pathogenesis of NS-related thrombotic risk is presented in Figure 1.

## 3. Clinical Predictors of Thrombotic Risk in Patients with NS

It is essential to keep in mind that many patients with NS may have other risk factors for venous thrombosis besides NS. Such risk factors include prolonged immobilization, recent surgery, prior DVT and PE, obesity, the presence of central venous catheters, stroke, and palsies [8]. Thus, it is essential to approach the evaluation of thrombotic risk in these patients in a thorough manner.

Several clinically useful predictors are of utility in stratifying patients with NS regarding the future risk of thrombotic events. First, histologic diagnosis of NS is of paramount importance in assessing the risk of thrombosis. Barbour et al. analyzed the data of 1,313 patients with idiopathic NS (395 subjects with MN, 370 subjects with FSGS, and 548 subjects with IgAN) [47] and demonstrated that the diagnosis of MN was associated with an increased risk of venous thromboembolism (VTE) compared to FSGS and IgAN (more than 2-fold increased risk compared to FSGS and more than 19-fold increased risk compared to IgAN). Lionaki et al. studied 898 patients with biopsy proven MN to assess possible predictors for increased thrombotic risk [48]. These investigators showed that an albumin level <2.8 g/L was independently associated with a higher thrombotic risk. Moreover, every 1.0 g/L reduction in albumin was translated into a 2.13-fold increased risk of VTE.

Causes of NS other than MN may impose a lesser risk for thrombosis, and, in such groups, a lower level of albumin may be associated with thrombotic risk. Cherng et al. demonstrated that patients with NS and an albumin level <2.0 g/L had a greater risk of VTE and PE [49]. It is essential to point out that no data on histological diagnosis were available in their study. Another interesting finding of their study was that 29% of patients had evidence of PE, with some of these cases being asymptomatic. Kuhlmann et al. also showed that an albumin level <2.0 g/L was associated with an increased thrombotic risk among patients with NS [50].

Age is an important risk factor for VTE among patients with NS since adult patients have an approximately 7- to 8-fold increased risk of a thrombotic event compared to children with NS [2, 51].

Therefore, based on the above data, it is essential to have a thorough approach for the analysis of thrombotic risk in patients with NS. Consideration of conventional risk factors for DVT and VTE is of paramount importance. Several specific clinical markers are of clinical use, such as a biopsy proven diagnosis of MN, albumin level <2.8 g/L in patients with MN, and albumin level <2.0 g/L in NS other than MN.

## 4. Clinical Presentation and Epidemiology of PE in Patients with NS

DVT is a major risk factor for PE [8]; lower extremities are the major site for DVT occurrence [52]. The most common symptoms of DVT are extremity swelling, erythema, and pain. Given its associated morbidity and mortality, it is essential to consider DVT in the differential diagnosis in patients who present with new onset extremity pain (especially when nontrauma related), swelling, and redness. Several clinical score systems are available to help clinicians stratify the risk of a possible DVT; Wells score for DVT is among the most commonly used ones [53] and is presented in Table 1. In patients with a low probability of DVT, a negative D-dimer test rules out DVT in most, but not in all patients [54]. In patients with a Wells score ≥1, Doppler ultrasound of lower extremities must be performed to exclude DVT [53]. In all patients with confirmed DVT, anticoagulation therapy is warranted and if medically contraindicated, an inferior vena cava (IVC) filter should be placed [52].

As mentioned above, DVT is a major risk factor for PE [8]. The clinical presentation of PE ranges from asymptomatic to nonspecific complaints of shortness of breath and chest pain to cardiovascular collapse and death [6]. A modified version of Wells score for PE is available for clinical use to help clinicians stratify the probability of PE [55]. Wells score for PE is presented in Table 2. A negative D-dimer in patients with a Wells score for PE ≤4 (some argue for ≤2) effectively excludes PE in most patients. Patients with a greater score or positive D-dimer should undergo computed tomography (CT) of the chest with the administration of intravenous contrast [8]. However, poor renal function (defined as elevated creatinine and/or decreased glomerular filtration rate), contrast allergy, and the simple lack of a CT machine may preclude this useful imaging modality in some patients. In such circumstances, ventilation-perfusion nuclear scan (V/Q) may be useful; a negative V/Q scan rules out PE and low probability of V/Q scan with a low clinical probability of PE is useful in excluding the disease; all other combinations generally require further testing. However, it is important to remember that V/Q scans have decreased sensitivity and specificity in patients with underlying pulmonary disease [10].

The management of PE is complex and depends on various factors, such as cardiovascular instability and the presence of right ventricular dysfunction. The reader is referred to focused reviews on this topic [8, 56].

DVT is the most common thrombotic complication of NS according to some studies. Kayali et al. studied 925,000 patients with NS and compared them to 898,253 patients without NS [57]. These researchers found that patients with NS had a greater risk for both DVT and PE, with a relative risk of 1.72 and 1.39, respectively. In contrast to them, Suri et al. showed that PE was more common than DVT (25.7 versus 16.6%, resp.) [58]. However, the study sample included only 34 pediatric patients with NS. This actually may explain the different findings in regard to the commonest thrombotic complication of NS.

It is important to keep in mind, however, that patients with NS also have an increased risk of renal vein thrombosis [57]. Therefore, in some patients with NS and PE, the origin of pulmonary embolus is from the renal veins [57].

Another important issue is that a considerable number of patients with PE and NS are asymptomatic (at least 12%), as demonstrated by Cherng et al. [49]. Thus, it is important to retain a clinical suspicion for VTE in patients with NS since even clinically silent and chronic PE may lead to a serious complication such as CTEPH [10].

## 5. Prophylaxis against Increased Thrombotic Risk in Patients with NS

It is essential to note that the scientific literature is scant, with no randomized data available on the topic of primary thrombotic prevention in patients with NS. Sarasin and Schifferli in their analysis demonstrated that patients with MN benefit from primary prophylactic anticoagulation [59]. Rostoker et al. studied 30 patients with NS to assess the utility of primary chemoprevention of thrombosis with low molecular weight heparin [60]. The study participants were followed for 13 months, and no thrombotic events or treatment side effects were observed. Nevertheless, given the small study sample, the lack of a control group, and the short follow-up period, it is impossible to translate their results into everyday clinical practice.

In a recent study, Lee et al. studied whether patients with MN benefit from primary prophylactic anticoagulation [61]. Patients were subdivided according to the levels of serum albumin into three groups: serum albumin <3.0 mg/dL, serum albumin <2.5 mg/dL, and serum albumin <2.0 mg/dL. As was discussed earlier in the text, patients with lower serum albumin (especially with serum albumin <2.0 mg/dL) represent a high risk group for thromboembolic events. Furthermore, these researchers stratified their bleeding risk using ATRIA score which was initially invented for patients with atrial fibrillation. It was demonstrated that patients who are at low risk of bleeding according to the ATRIA score would benefit from anticoagulation (benefit to risk ratio of 4.5 : 1, 5.2 : 1, and 13.1 : 1 for patients with serum albumin < 3.0 mg/dL, serum albumin <2.5 mg/dL, and serum albumin <2.0, resp.). Patients who are at intermediate risk of bleeding according to the ATRIA score and serum albumin level <2.0 mg/dL were found to have the benefit to risk ratio of 3.9 : 1 and patients with high bleeding risk should not be treated with prophylactic anticoagulation according to their results given high risk of serious bleeding (online calculator is available at http://www.unckidneycenter.org/gntools/gntools-team.html to calculate the benefit to risk ratio of anticoagulation according to their data). However, patients with hypoalbuminemia may be at increased risk of bleeding and, indeed, this parameter is a part of ATRIA score. However, this is unclear whether hypoalbuminemia is a true risk factor for bleeding in patients with NS (ATRIA score is primarily for patients with atrial fibrillation). The other limitations of this study are its retrospective analysis, no control group, and inclusion of patients only with MN. Therefore, the approach above cannot be recommended to patients with NS histology other than MN.

Medjeral-Thomas et al. retrospectively studied different thromboprophylactic regimens in 143 patients with NS (58 with MN, 45 with MCD, and 40 with FSGS) [62]. Patients were stratified according to their serum albumin levels (serum albumin >3.0 mg/dL, serum albumin 2-3 mg/dL, and serum albumin <2.0 mg/dL). Patients with serum albumin > 3.0 mg/dL did not receive any prophylactic anticoagulation, patients with serum albumin 2.0-3.0 mg/dL received low dose aspirin (75 mg/day), and patients with serum albumin < 2.0 mg/dL received either low dose of low molecular weight heparin or warfarin with a goal international normalized ratio of 1.5–2.5. Two patients developed venous thrombotic events within a week of starting anticoagulation, one patient on anticoagulation developed urgent gastrointestinal bleeding, and two patients on anticoagulation received elective blood transfusion for occult gastrointestinal bleeding. The major limitations of this study are its retrospective design and lack of the control group.

The question is, therefore, what can be done until solid data are available? First, it is essential to discuss the pros and cons of anticoagulation in all patients with NS (especially in patients with hypoalbuminemia), which should lead to informed patient decisions. Second, it is prudent to state that NS secondary to MN may impose a greater thrombotic risk. Third, other risk factors for DVT, VTE, and PE should be considered (e.g., a recent surgery or prior DVT/PE), and pharmacological anticoagulation should be commenced, unless being contraindicated. Fourth, it is important to consider compelling indications for pharmacological anticoagulation, such as atrial fibrillation. Fifth, statins, which are commonly used in patients with NS, may modestly decrease the risk of DVT [63]. Sixth, aspirin may be used to diminish the risk of VTE, as shown in recent studies published in the New England Journal of Medicine [64] and Clinical Journal of the American Society of Nephrology [62]. However, it is essential to mention that there are no data from studies of the utility of aspirin for thromboprophylaxis in patients with NS.

A much clearer situation is when the patient is found to have a thrombotic event, such as DVT, renal vein thrombosis, or PE. In such cases, active therapeutic pharmacological anticoagulation is warranted, unless being contraindicated [8]. In cases of PE with cardiovascular compromise and right ventricular dysfunction, which is also known as a massive PE, there is a need for pharmacological thrombolysis or embolectomy if thrombolysis is contraindicated [56]. The data regarding elastic compression stockings and their impact on the incidence of VTE is lacking, but, given a low adverse effect profile, this approach may be recommended for some patients with NS who are at an increased risk of DVT and for patients with clear contraindications to anticoagulation [65]. It is important to note that the treatment approach is not different for patients with NS and the reader is referred to some well-written reviews on this topic [6, 66].

In patients with DVT who are not candidates for pharmacological anticoagulation, IVC filters should be placed [66]. Suprarenal IVC filters may be suitable for patients with NS given a greater incidence of renal vein thrombosis in this group [67]. It is important to keep in mind that IVC filters are associated with some complications, though being found in a small number of patients [68–70]. The list of potential complications of IVC filter insertion includes thrombotic complications such as thrombosis at the site of insertion, local complications such as hematoma formation at the insertion site, and filter associated complications such as filter migration, filter embolization, and erosion of the IVC.

Another question, which urgently needs prospective randomized data, is regarding the duration of anticoagulation (either for primary or secondary prevention and treatment) in patients with NS. A common belief is that patients with NS who are at remission for at least 2 years have decreased thrombotic risk. Indeed, as was discussed above, patients with normal or near normal serum albumin levels have minimal risk of thrombosis and do not benefit from pharmacological prophylactic anticoagulation. Another factor to consider is whether NS is secondary to cancer since the latter may further increase the risk of thrombosis [11].

We suggest that the overall risk assessment is essential and will provide some possible examples which clinicians may encounter in everyday clinical practice. The first theoretical case was a 35-year-old nonobese male with NS secondary to FSGS, without any additional risk factors for DVT and no history of a thrombotic event and albumin level of 2.1 g/L. When approaching such a case, it is important to keep in mind that FSGS is thought to be less associated with DVT/VTE than NS secondary to MN. An albumin level ≤2.0 g/L has been found to impose increased thrombotic risk in such patients (discussed in previous sections). Therefore, in such cases, it is essential to educate the patient on the condition and its potential thrombotic risk, benefits versus risks of anticoagulation, and any ancillary therapies which might modestly reduce thrombotic risk (such as statins and low dose aspirin). Furthermore, it should be clearly conveyed to the patient that there is no randomized prospective data regarding anticoagulation in patients with NS and FSGS in particular. Thus, a patient's informed decision is the key in such a scenario; however, from the current evidence standpoint, we would not start this patient on pharmacological anticoagulation but may offer low dose aspirin for thromboprophylaxis if he has no contraindications to antiplatelet therapy.

The second theoretical case was a 47-year-old female with NS secondary to MN, an albumin level of 1.7 g/l, congestive heart failure, and atrial fibrillation. This case is relatively straightforward since the patient has many compelling indications for pharmacological anticoagulation, such as atrial fibrillation and an albumin level of 1.7 in a setting of MN. Furthermore, the presence of atrial fibrillation obviates the need to consider serum albumin in this patient. Therefore, such patients should be counseled on their substantially increased risk of a thrombotic event (either venous or embolic stroke). The third theoretical case was a 50-year-old female with NS secondary to MCD, with no compelling indications for thromboprophylaxis, risk factors for DVT, or prior history of DVT and an albumin level of 1.7 g/L. In this case, the patient should be counseled that based on the albumin level she is probably at increased risk for DVT. However, randomized scientific data are lacking and the decision of whether to anticoagulate or not is the most challenging in this particular case. One may consider extrapolating the approach used by Lee et al. [61] and estimate the bleeding risk using ATRIA score. However, it is essential to remember that study by Lee did not include patients with MCD. Therefore, detailed patient education and mutual decision-making are especially important in this case.

Finally, it is essential to address the question regarding the duration of anticoagulation. Again, there are no data on how long anticoagulation should be continued in patients with NS. One potential approach is to continue anticoagulation (if no contraindication) till serum albumin levels normalize and the patients with NS achieve remission. Nevertheless, we believe that overall and thorough risk factor assessment is essential. For example, we will use indefinite anticoagulation (unless contraindicated) in cases of NS with low albumin levels and a compelling indication for anticoagulation such as atrial fibrillation or multiple VTE.

## 6. Conclusion

Patients with NS are at an increased risk of thrombotic events; DVT, RVT, and PE are quite common in patients with NS. It is important to note that NS secondary to MN may impose a greater thrombotic risk for unclear reasons. Increased platelet activation, enhanced RBC aggregation, and an imbalance between procoagulant and anticoagulant factors are thought to underlie the excessive thrombotic risk in patients with NS. The current scientific literature does not provide a solid answer on the role of primary thromboprophylaxis among patients with NS. One possible exception is patients with MN and hypoalbuminemia who are at low risk for bleeding. A thorough approach which includes accounting for all additional thrombotic risk factors is, therefore, essential. Patient counseling regarding the pros and cons of anticoagulation is of paramount importance. Future studies should address the question regarding the utility of primary thromboprophylaxis.

## Figures and Tables

**Table 1:** Wells score for DVT (adapted from [52]).

Variable	Points
Active cancer (treatment ongoing or within the previous 6 months or palliative treatment)	1
Paralysis, paresis, or recent immobilization of the lower extremities	1
Recently bedridden for 3 days or more or major surgery within previous 12 weeks requiring general or regional anesthesia	1
Local tenderness along the distribution of the deep venous system	1
Entire leg swollen	1
Calf swelling >3 cm compared to asymptomatic leg (measuring 10 cm below tibial tuberosity)	1
Pitting edema confined to the symptomatic leg	1
Nonvaricose collateral veins	1
Previously documented DVT	1
Alternative diagnosis at least as likely as DVT	−2

Scoring:

<0—low pretest probability.

1-2—moderate pretest probability.

≥3—high pretest probability.

**Table 2:** Wells score for PE (adapted from [54]).

Variable	Points
Clinical signs and symptoms compatible with DVT	3
PE judged to be the most likely diagnosis	3
Surgery or bedridden for more than 3 days during the past 4 weeks	1.5
Previous DVT or PE	1.5
Heart rate > 100/minute	1.5
Hemoptysis	1
Active cancer (treatment ongoing or within the previous 6 months or palliative treatment)	1

Scoring:

≤4—low pretest probability.

4.5–6—moderate pretest probability.

>6—high pretest probability.
